# Dual Mobility vs Constrained Liners in Managing Instability After Revision Total Hip Arthroplasty: A Systematic Review and Exploratory Cost-Effectiveness Model

**DOI:** 10.7759/cureus.108186

**Published:** 2026-05-03

**Authors:** Arfaz Shaik, Nimra Akram, Mustafa Al-Saadi, Umar Hayat, Irrum Afzal, Sarkhell Radha

**Affiliations:** 1 Orthopaedics and Trauma, Croydon University Hospital, Croydon Health Services NHS Trust, London, GBR; 2 Orthopaedics, Stoke Mandeville Hospital, Buckinghamshire Healthcare NHS Trust, Aylesbury, GBR; 3 Orthopaedics, Medway Maritime Hospital, Medway NHS Foundation Trust, Gillingham, GBR; 4 Orthopaedics, Croydon University Hospital, Croydon Health Services NHS Trust, London, GBR

**Keywords:** constrained liners, dislocations, dual-mobility, instability, meta-analysis, re-revision, revision tha, survivorship

## Abstract

Recurrent dislocation following revision total hip arthroplasty (THA) remains a major clinical challenge. Dual-mobility (DM) constructs and constrained liners (CLs) are commonly used to improve stability; however, direct comparative evidence remains limited. A systematic review was conducted in accordance with Preferred Reporting Items for Systematic Reviews and Meta-Analyses (PRISMA) guidelines. Multiple databases were searched from January 2000 to October 2025 for studies comparing DM constructs and CLs in revision THA. The primary outcomes were dislocation and aseptic re-revision, while secondary outcomes included implant survivorship, patient-reported outcome measures (PROMs), patient- and construct-related risk factors, and non-revision complications. Due to heterogeneity in study designs, results were synthesized descriptively. Ten comparative observational studies comprising 2,046 revision hips (1,237 DM and 809 CL) were included. DM constructs were associated with lower reported dislocation and aseptic re-revision rates compared with CLs. Five-year re-dislocation-free survivorship ranged from 78% to 85% across comparative cohorts. In registry-based analyses, the 10-year cumulative aseptic re-revision rate was 8.6% for DM and 13.1% for CLs. PROMs, assessed using the modified Harris Hip Score (mHHS), improved similarly in both groups. No included comparative study reported primary cost or cost-utility data. However, external economic models suggest potential cost-effectiveness advantages with DM constructs. These benefits were driven primarily by lower complication rates and fewer re-revisions. In revision THA, DM constructs are consistently associated with lower postoperative instability compared with CLs in comparative observational studies. Evidence regarding reductions in aseptic re-revision is heterogeneous, reflecting competing failure mechanisms and the limitations of non-randomized data. Economic implications remain uncertain due to the absence of primary cost-utility analyses. Implant selection should therefore be individualized, and high-quality prospective comparative studies with standardized outcome reporting are needed to define optimal construct selection in revision THA.

## Introduction and background

Revision total hip arthroplasty (THA) addresses failed primary constructs but is frequently complicated by postoperative instability, with reported dislocation rates of 10-35%, compared with 1-5% after primary THA [1-3]. Dislocation following revision THA is associated with significant functional impairment, an increased risk of subsequent re-revision, and higher healthcare utilization and costs. To mitigate instability, surgeons commonly use dual-mobility (DM) constructs or constrained liners (CLs); however, direct comparative evidence regarding their effectiveness and durability remains limited [4,5].

DM constructs increase effective head size and jump distance through a dual-articulation mechanism, whereas CLs provide stability via mechanical containment within a locking system. These differing designs are associated with distinct failure mechanisms. CLs may be prone to liner dissociation, locking-ring failure, and increased stress transfer to the bone-implant interface. At the same time, DM constructs carry risks, including intraprosthetic dislocation and polyethylene wear [6,7]. Despite these limitations, CLs remain widely used due to long-standing clinical familiarity and variations in implant acquisition costs across healthcare systems [8].

The choice between DM constructs and CLs in revision THA has important clinical implications. Although DM constructs are associated with higher initial implant costs, potential reductions in instability-related complications and reoperations may enhance overall value; however, cost-effectiveness data specific to revision THA remain limited [9,10]. Previous systematic reviews have primarily evaluated single-arm cohorts or pooled incidence rates by implant type, limiting direct comparisons [7,11]. Moreover, such approaches often fail to account for differences in surgical indications and case complexity. Therefore, the present review focuses exclusively on studies that directly compare DM constructs and CLs within the same study populations to better inform implant selection and instability risk.

We hypothesize that DM constructs are superior to CLs in revision THA and should be considered first-line options in most revision settings. In contrast, CLs may be reserved for salvage situations involving poor soft-tissue envelope, multiple prior revisions, or failed DM constructs. This review evaluates the available evidence supporting this hypothesis.

The primary objective of this study was to compare DM constructs and CLs in revision THA, focusing on instability-related outcomes, including dislocation and aseptic re-revision. Secondary outcomes included implant survivorship, complications, and patient-reported outcome measures (PROMs). We also examined patient- and construct-related factors influencing instability, including age, body mass index (BMI), abductor status, and femoral head size. In addition, potential economic implications were explored using published cost and utility estimates. By synthesizing contemporary comparative evidence, this study aims to clarify areas of consensus and uncertainty in the literature and to identify priorities for future prospective research.

## Review

Methodology 

Search Strategy

This systematic review and meta-analysis were conducted in accordance with the Preferred Reporting Items for Systematic Reviews and Meta-Analyses (PRISMA) 2020 guidelines and prospectively registered with the International Prospective Register of Systematic Reviews (PROSPERO) (registration number: CRD420251062775). A comprehensive literature search was performed in PubMed, Embase, Scopus, Web of Science, and the Cochrane Library from January 2000 to October 2025. Grey literature was screened using Google Scholar. Detailed search strategies for all databases are provided in Appendix A.

Study Selection

Studies were eligible if they included adults (≥18 years) undergoing revision THA and directly compared DM constructs with CLs. Eligible studies were required to report extractable data for at least one primary outcome. Randomized controlled trials and comparative observational studies with a minimum follow-up of 12 months were included.

Studies were excluded if they were non-comparative, involved only primary THA, lacked extractable outcome data, or were case reports, narrative reviews, or biomechanical studies.

**Figure 1:**
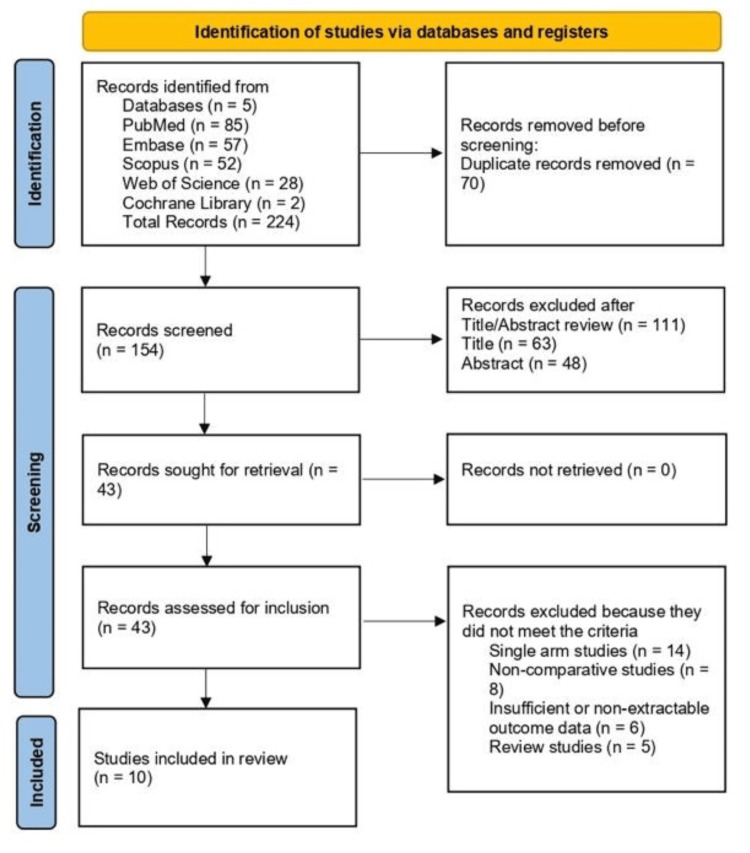
PRISMA flow diagram illustrating study selection for the systematic review and meta-analysis PRISMA: Preferred Reporting Items for Systematic Reviews and Meta-Analyses

Two reviewers independently screened titles and abstracts, followed by full-text review, using Covidence (Veritas Health Innovation Ltd, Melbourne, Australia). Disagreements were resolved through consensus or by adjudication from a third reviewer. Duplicate records were removed using EndNote X9 (Clarivate Plc, London, United Kingdom). The study selection process is summarized in a PRISMA flow diagram (Figure 1).

Data Extraction

Data were independently extracted by two reviewers using a standardized electronic data collection form. Extracted variables included study characteristics (author, year, study design, sample size, and follow-up duration), patient demographics, and outcomes.

The primary outcomes were dislocation and aseptic re-revision. Secondary outcomes included implant survivorship, PROMs, and non-revision complications. Non-revision complications were defined a priori as adverse events not requiring re-revision surgery, including deep infection managed non-operatively, aseptic loosening not requiring revision, periprosthetic fracture, liner or locking-mechanism failure, adverse local tissue reactions, and other construct-related complications.

Secondary outcomes were synthesized descriptively due to heterogeneity in outcome definitions, follow-up durations, and reporting formats. Given the limited availability of cost data, a narrative synthesis incorporating external economic models that estimate the cost per quality-adjusted life year (QALY) and reoperation-related savings was conducted.

Methodological Quality and Certainty of Evidence

The methodological quality of included studies was assessed using the Methodological Index for Non-Randomized Studies (MINORS). For comparative studies, the maximum score is 24, with each item rated on a scale of 0 to 2 (0 = not reported, 1 = reported but inadequate, 2 = reported and adequate). Scores of 0-12 were considered low quality, 13-18 moderate quality, and >18 high quality. Assessments were conducted independently by two reviewers, with discrepancies resolved through consensus.

The certainty of evidence for key outcomes was evaluated using the Grading of Recommendations Assessment, Development and Evaluation (GRADE) approach. Observational studies were initially rated as low certainty and could be downgraded for risk of bias, inconsistency, indirectness, imprecision, or publication bias, or upgraded for large effect sizes. Certainty ratings were categorized as high, moderate, low, or very low.

Data Analysis

Outcomes were summarized descriptively at the study level. Sensitivity analyses were not performed due to the limited number of included studies.

Results

Study Selection and Characteristics

Ten observational comparative studies [12-21] were included in the qualitative synthesis, comprising 2,046 revision hips: 1,237 DM hips and 809 CL hips. The studies were published between 2000 and 2025 and included six retrospective cohort studies [12-17] and four registry-based retrospective analyses [18-21]. Among retrospective cohorts, mean follow-up ranged from 24 to 60 months, whereas registry-based analyses reported follow-up of up to 10-11 years.

Implant selection was non-randomized in all studies. Reporting of baseline characteristics and revision indications was variable. In one institutional cohort, baseline demographics and intraoperative factors were comparable between DM and CL groups [16]. In contrast, other cohorts showed differences in follow-up duration, treatment era [13], or prevalence of abductor deficiency [15]. Study designs and baseline characteristics are summarized in Table 1.

**Table 1:** Characteristics of included studies and role in evidence synthesis Note: In multi-arm cohort studies, only dual mobility and constrained liner arms were extracted CL, constrained liner; DM, dual-mobility; n, number of hips; THA, total hip arthroplasty; NR, not reported.

Study	Design	Data source	Mean Follow-up	Comparator	DM (n)	CL (n)	Outcomes reported	Key Finding (DM vs CL)
Stevenson et al. (2020) [12]	Retrospective cohort (multi-arm)	Single-centre	4.7 years	DM vs CL arm extractable	48	11	Dislocation; aseptic re-revision	Lower complication burden reported with DM
Efimov et al. (2018) [13]	Retrospective cohort	Single-centre	1.5 - 5.5 years	DM vs CL	58	78	Dislocation; aseptic re-revision	Lower cumulative aseptic re-revision probability reported with DM
Klemt et al. (2020) [14]	Retrospective cohort (multi-arm)	Single-centre	4 years	DM vs CL arm extractable	42	24	Dislocation	Lower instability-related failure reported with DM
Scholz et al. (2024) [15]	Retrospective cohort (multi-arm)	Single-centre	5 years	DM vs CL arm extractable	38	9	Dislocation	Lower re-revision risk reported with DM
Chisari et al. (2022) [16]	Retrospective cohort	Single-centre	14.3 months	DM vs CL	139	52	Dislocation; aseptic re-revision	Lower risk of second revision reported with DM
Valenzuela et al.(2022) [17]	Retrospective cohort	Single-centre	13.2 months (DM)/50.0 months (CL)	DM vs CL	25	25	Dislocation; re-revision	Both constructs had similar successful outcomes
Hoskins et al. (2020) [18]	Registry	AOANJRR	2.4 - 4.9 years	DM vs CL	265	288	Aseptic re-revision	Low instability in both constructs
Khatod et al. (2024) [19]	Registry	Kaiser Permanente	Up to 10 years	DM vs CL	375	268	Aseptic re-revision	No clear difference; limited power
Stučinskas et al. (2018) [20]	Registry (nationwide)	Lithuanian Arthroplasty Register	2 years	DM vs CL	247	54	Dislocation; re-revision	Similar functional outcomes: instability reduced with DM
Hermansen et al. (2021) [21]	Registry-based retrospective cohort	Danish Hip Arthroplasty Register	Median 5.3 years	DM vs CL	NR	NR	Dislocation; re-revision; risk-factor analysis	Both constructs were associated with lower dislocation rates

Methodological Quality and Certainty of Evidence

MINORS scores ranged from 16 to 20 out of 24, indicating moderate to good methodological quality and a low risk of bias across predominantly retrospective studies. According to the GRADE approach, the certainty of evidence was moderate for dislocation outcomes and low to moderate for aseptic re-revision, implant survivorship, and complications. These ratings reflect consistent effect directions across institutional and registry-based studies despite their non-randomized designs (Table 2).

**Table 2:** Grade assessment of certainty of evidence for dual-mobility versus constrained liners in revision total hip arthroplasty GRADE, Grading of Recommendations Assessment, Development and Evaluation; THA, total hip arthroplasty

Outcome	Studies	Risk of Bias	Inconsistency	Indirectness	Imprecision	Certainty
Dislocation	8	Moderate	Moderate	Moderate	Moderate	Moderate
Aseptic re-revision	8	Moderate	Low	Moderate	Moderate	Low to Moderate
Complications	5	Moderate	Low	Moderate	Low	Low to Moderate
Implant Survivorship	5	Moderate	Moderate	Low	Moderate	Low to Moderate

Inconsistent reporting of risk factors introduced potential selection bias, particularly in smaller cohorts [17]. Egger’s regression indicated no significant publication bias (dislocation: intercept −1.30, p = 0.48; re-revision: intercept −1.07, p = 0.294). Sensitivity analyses showed that no single study disproportionately influenced the results. Moderate heterogeneity was observed, likely attributable to variations in surgical technique, follow-up duration, and patient comorbidities.

Dislocation Outcomes

**Figure 2:**
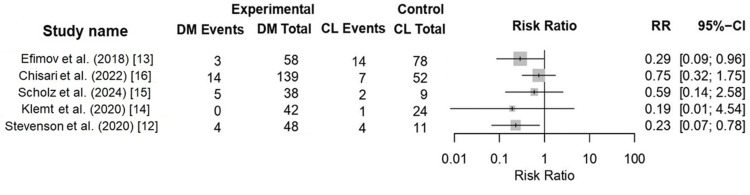
Forest plot comparing dislocation risk following revision total hip arthroplasty using dual-mobility (DM) constructs versus constrained liners (CL). References: [12-16]

Eight studies reported dislocation outcomes (n = 1,996 hips: 1,212 DM and 784 CL) [12-17,20,21]. Five studies provided extractable comparative data (Figure 2), while the remaining studies reported outcomes descriptively. DM constructs were associated with a lower risk of dislocation compared with CLs (8.0%, 26/325 vs. 16.1%, 28/174). Registry-based analyses also demonstrated lower adjusted instability rates with DM constructs; however, follow-up duration, adjustment methods, and revision indications varied across datasets.

Aseptic Re-Revision

**Figure 3:**
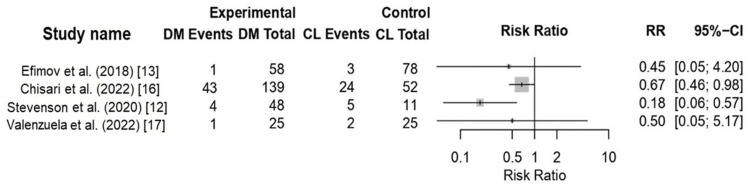
Forest plot comparing aseptic re-revision risk following revision total hip arthroplasty using dual-mobility (DM) constructs versus constrained liners (CL) References: [12,13,16,17]

Eight studies reported aseptic re-revision outcomes (n = 1,933 hips: 1,157 DM and 776 CL) [12,13,16-21]. Four studies provided comparative event counts suitable for graphical presentation (Figure 3). DM constructs were associated with a lower risk of re-revision. Recurrent instability was the most common indication for re-revision in both groups. Liner-related failures, including dissociation and locking-mechanism failure, were reported exclusively in CL cohorts in studies that differentiated failure mechanisms [12,16,19]. However, detailed reporting of re-revision indications was inconsistent.

Time-to-Event and Survivorship Outcomes

Three registry studies reported hazard ratios for aseptic re-revision using time-to-event analyses [18-20], adjusting for patient-, implant-, and surgeon-level covariates. Institutional cohorts reported crude event rates without multivariable adjustment. Across studies, hazard ratios for aseptic re-revision were consistently below 1.0 for DM constructs; however, formal pooling was not performed due to methodological heterogeneity.

Implant survivorship, reported in five studies [14,15,18-20], was assessed using Kaplan-Meier analyses with follow-up ranging from two to 11 years. Institutional cohorts reported five-year re-dislocation-free survivorship estimates of 78-85%, whereas registry-based analyses reported cumulative 10-year aseptic re-revision rates (Table 3). Incomplete implant-specific reporting and variable follow-up durations precluded formal pooled survivorship analysis.

**Table 3:** Implant survivorship outcomes for dual-mobility versus constrained liners in revision total hip arthroplasty CL, constrained liner; DM, dual-mobility; KM, Kaplan–Meier; THA, total hip arthroplasty; ALTR, adverse local tissue reaction

Study	Timepoint	Survivorship endpoint	DM outcome	CL outcome	Comparator Notes
Klemt et al. (2020) [14]	3 years	Implant survivorship (Kaplan–Meier)	87%	74%	ALTR cohort with severe abductor deficiency
Scholz et al. (2024) [15]	5 years	Re-dislocation–free survivorship (Kaplan–Meier)	85%	78%	Direct DM vs CL comparison; institutional cohort
Hoskins et al. (2020) [18]	11 years	Cumulative second revision (all constructs)	Not reported	Not reported	Implant-specific DM vs CL survivorship percentages not extractable. Cumulative re-revision rates are 24% for entire cohort
Khatod et al. (2024) [19]	10 years	Cumulative aseptic re-revision probability	8.6%	13.1%	Registry study; DM vs CL reported explicitly
Stucinskas et al. (2018) [20]	≤5 years	Cumulative re-revision for dislocation	2.4%	10.3%	Comparator = “other constructs” (includes constrained cups; not CL-only)
		Cumulative re-revision for any reason	8.2%	19.7%	Same mixed comparator group (not CL-only)

Risk Factors and Patient-Reported Outcomes

Age and BMI did not demonstrate a consistent association with dislocation or aseptic re-revision outcomes across included studies [13,15,16,21] (Table 4). Abductor deficiency was frequently reported in instability cohorts and, where stratified, was more common in CL groups. In patients with compromised soft-tissue function, DM constructs achieved stability comparable to CLs [14,17].

**Table 4:** Patient- and construct-related risk factors reported in revision total hip arthroplasty studies BMI, body mass index; CL, constrained liner; DM, dual-mobility; RR, risk ratio; NR, not reported; sHR, sub-distribution hazard ratio

Study	Mean age (DM | CL)	Mean BMI (DM | CL) (kg/m²)	Sex (DM | CL) (%)	Femoral head size (mm)	Abductor deficiency	Risk factor analysis & key interpretation
Stevenson et al. (2020) [12]	61.0 years	Obesity (BMI ≥35) reported	Not reported	≤32 mm vs ≥36 mm; DM vs CL	Partial (14.2% of instability cases)	Descriptive cohort only; dislocation rates reported by head size and construct without patient-level modelling
Efimov et al. (2018) [13]	59.9 | 58.1 years	29.94 | 28.38	Not reported	28 mm vs 32 mm	Not reported	Descriptive analysis: 28-mm heads associated with higher dislocation risk (RR 4.97); no multivariable adjustment
Klemt et al. (2020) [14]	55 | 71 years	28 | 28	Not reported	Not reported	100% cohort	Restricted high-risk cohort: acceptable survivorship despite severe abductor deficiency
Scholz et al. (2024) [15]	73.8 | 65.2 years	26.3 | 27.5	Not reported	Larger heads predominantly with DM	DM: 58% | CL: 89%	Group and subgroup analysis: abductor deficiency strongly associated with instability; CL cohort had higher prevalence
Chisari et al. (2022) [16]	61.6 | 64.8 years	27.6 | 28.7	Men 44.9 | 33.3, Women 55.1 | 66.7	Not reported	Not reported	Descriptive comparison only; no formal patient- or construct-level risk modelling
Valenzuela et al. (2022) [17]	75 | 73 years	28.8 | 31.6	Men 52.0 | 48.0	NR	More common in CL/TC group; not quantified by arm	Descriptive comparison only; no formal multivariable modelling. DM is favoured except in global abductor deficiency or severe joint laxity
Hoskins et al. (2020) [18]	72.2 | 74.9 years	Not reported	Not reported	Not reported	Not reported	Registry-based multivariable analysis: increasing age increased risk of second revision; implant type independently predictive
Khatod et al. (2024) [19]	67.8 | 73.7 years	29.4 | 27.2	Men 33.1 | 29.1	Not reported	Not reported	Multivariable Cox regression: older age and higher BMI increased aseptic re-revision risk; implant articulation remained independently associated
Stucinskas et al. (2018) [20]	72 | 73 years	Not reported	Not reported	Not reported	Not reported	Cox regression adjusted for age and sex: dual mobility associated with lower re-revision risk
Hermansen et al. (2021) [21]	NR	NR	NR	Head size analysed, but not reported separately for DM and CL	NR	Multivariable competing-risk regression: CL reduced dislocation risk (sHR 0.36, 95% CI 0.27–0.48) and DMC reduced dislocation risk (sHR 0.21, 95% CI 0.08–0.58) versus regular liners; head/liner-only exchange increased risk of dislocation and re-revision

Femoral head size demonstrated a consistent effect: smaller head sizes were associated with a higher risk of instability regardless of articulation type. In contrast, constructs with larger effective head diameters showed lower dislocation rates [12,13]. PROMs improved postoperatively in both groups, with no consistent or clinically meaningful differences between DM and CL constructs.

Complications

Non-revision complications were variably reported and synthesized descriptively (Table 5). In one cohort, deep infection, aseptic loosening, and liner or locking-mechanism failure were numerically higher in CL constructs [13]. In patients with severe abductor deficiency, periprosthetic fracture and adverse local tissue reactions were low and comparable between groups [14]. One institutional series reported higher overall non-revision complication rates in CL constructs [16], whereas another cohort observed aseptic loosening only in DM constructs [12]. Other reported reoperations included sterile hematoma, infection, and insert exchange, with no cup loosening observed in either group [17].

**Table 5:** Non-revision complication rates for dual-mobility and constrained-liner constructs following revision total hip arthroplasty ALTR, adverse local tissue reaction; CL, constrained liner; DM, dual-mobility; N, number of hips; THA, total hip arthroplasty

Study	Complication	DM (events/N, %)	CL (events/N, %)
Stevenson et al. (2020) [12]	Aseptic loosening	3/48 (6.3)	0/11 (0)
Efimov et al. (2018) [13]	Deep infection	4/58 (6.9)	8/78 (10.3)
	Aseptic loosening	1/58 (1.7)	3/78 (3.8)
	Liner/locking-mechanism damage	1/58 (1.7)	4/78 (5.1)
Klemt et al. (2020) [14]	Periprosthetic fracture	1/42 (2.4)	1/24 (4.2)
	Recurrent adverse local tissue reaction (ALTR)	2/42 (4.8)	1/24 (4.2)
	Other complications (composite; author-defined)	2/42 (4.8)	1/24 (4.2)
	Iliopsoas tendonitis (non-operative)	3/43 (7.0)	Not reported
Chisari et al. (2022) [16]	Intrahospital complication (composite)	15/72 (20.8)	8/25 (32.0)
	Readmission within 90 days	28/139 (20.1)	9/52 (17.3)
Valenzuela et al. (2022) [17]	Acute infection requiring reoperation	0/25 (0)	1/25 (4)
	Sterile haematoma requiring reoperation	2/25 (8%)	1/25 (4)
	Revision for wear/liner failure	0/25 (0)	2/25 (8)
	Periprosthetic femur fracture	1/25 (4)	0/25 (0)
	Cup loosening / cement-fixation failure	0/25 (0)	0/25 (0)

Cost-Effectiveness Analysis

No included study reported primary cost or cost-utility data. External economic models suggest that DM implants, despite higher upfront costs (average $5,726), provide long-term economic benefits [22]. A French model estimated that DM use in 100,000 THAs would generate 793,086 QALYs, 441 more than with fixed-bearing implants, and save €28.3 million (£22.8M; $31.1M) in downstream costs [23].

**Figure 4:**
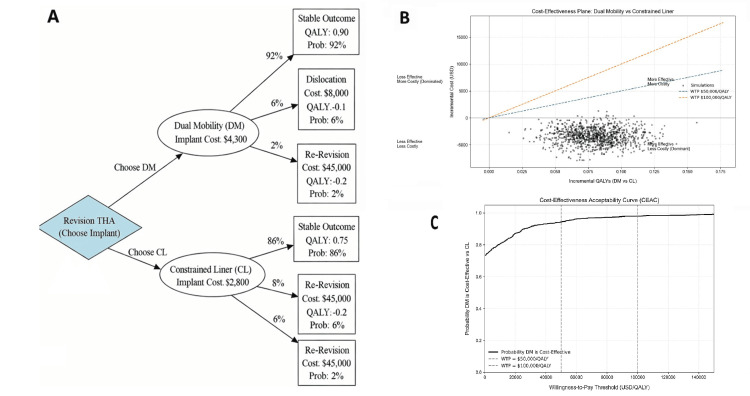
Exploratory Markov model, cost-effectiveness plane, and acceptability curve comparing dual-mobility (DM) constructs and constrained liners (CL) in revision total hip arthroplasty. (A) llustrative decision tree depicting revision total hip arthroplasty pathways for DM constructs and CL, based on pooled clinical outcomes and published cost and utility assumptions. (B) Cost-effectiveness plane showing incremental costs and quality-adjusted life years (QALYs) for DM versus CL across probabilistic simulations (C) Cost-effectiveness acceptability curve (CEAC) illustrating the probability of DM being cost-effective across a range of willingness-to-pay thresholds

Although CLs remain cost-effective in high-risk patients, DM constructs may offer greater value in younger or more active individuals by reducing complications and reoperations [9,24]. A Markov model using transition probabilities derived from the included studies evaluated cost-effectiveness from a healthcare system perspective (Figure 4; Appendix B). DM constructs were associated with higher utility (0.80 vs. 0.75 QALYs for DM and CL, respectively) and a 78% probability of cost-effectiveness at a $50,000 willingness-to-pay threshold. Sensitivity analyses identified CL dislocation risk and DM implant cost as the primary drivers of the model. DM remained cost-effective across plausible parameter ranges and reduced long-term per-patient costs by avoiding reoperations (DM dislocation: 6-14%; CL dislocation: 10-20%; DM cost: $4,000-$7,000 (£3,200-£5,600)).

Discussion

The findings of this review support existing evidence demonstrating lower postoperative instability with DM constructs compared with CLs, even in high-risk revision THA settings [11,18,19,21,25-27]. This consistency across study designs, healthcare systems, and revision indications aligns with the proposed biomechanical advantages of dual articulation, including increased jump distance and reduced impingement.

An important consideration in interpreting comparative outcomes is the clinical context of implant selection. DM constructs are typically used when intraoperative stability can be achieved with modular articulation. In contrast, CLs are often reserved for cases in which stability cannot be achieved despite optimized component positioning and soft-tissue tension [28,29]. This confounding by indication may partially explain observed differences in instability rates and highlights that implant selection often reflects underlying case complexity rather than purely device performance.

Large registry-based analyses provide robust contextual support, demonstrating a reduced risk of dislocation with DM constructs after adjustment for demographic and procedural factors [19]. Similar findings across national arthroplasty datasets reinforce the generalizability of this stability benefit [20,24,25]. These studies acknowledge a preference for DM constructs in patients at higher baseline risk of instability, including those with prior recurrent dislocations, severe abductor deficiency, retained well-fixed acetabular components, multiple prior revisions, neuromuscular comorbidities, or compromised soft-tissue envelopes. Despite adjustment, residual confounding cannot be excluded.

Evidence regarding aseptic re-revision is more nuanced. Several meta-analyses and institutional series report numerically lower re-revision rates with DM constructs compared with CLs [11,25,26]. However, high-quality registry-based analyses suggest that the dislocation benefit of DM constructs does not always translate into proportional reductions in overall re-revision risk [16,18,21]. For example, a large United States registry cohort found no significant long-term difference in aseptic re-revision risk between DM and CL after multivariable adjustment [19], suggesting that improved early stability does not necessarily translate into superior implant durability.

The divergence between instability and survivorship outcomes reflects differing failure mechanisms. CLs, while mechanically effective in limiting motion, may increase stress transfer to the bone-implant interface and are associated with liner dissociation, shell loosening, and mechanical failure in mid- to long-term follow-up (2-5 years; up to 10-11 years) [30-32]. Conversely, DM constructs introduce risks such as intraprosthetic dislocation; however, systematic reviews indicate that this complication remains uncommon in modern designs, particularly with highly cross-linked polyethylene [10,33,34]. Consequently, competing failure mechanisms may offset early gains in stability and influence patterns of early and late revision.

Patient- and construct-related modifiers provide further context for observed differences in stability. Demographic factors such as age and BMI do not appear to meaningfully modify the relationship between articulation type and postoperative instability [16,18,19], suggesting limited utility in guiding construct selection. In contrast, soft-tissue competence, particularly abductor function, plays a central role in postoperative stability, influencing both implant selection and outcomes. Neither construct reliably eliminates instability in the presence of global soft-tissue deficiency, highlighting the need for individualized articulation choice based on patient-specific risk [14,15]. Limited data exist on DM use following prior CL failure [35], preventing generalizable recommendations in this context.

Femoral head size emerges as an important modifier of instability risk in revision THA. Smaller head sizes are associated with greater instability irrespective of articulation type, whereas larger effective head diameters confer greater stability [12,13,36-38]. These findings underscore the multifactorial nature of instability and reinforce the importance of considering articulation choice alongside head size, component positioning, and soft-tissue integrity rather than as a standalone intervention.

Patient-reported outcomes remain limited. Where reported, functional scores, such as the Harris Hip Score, generally improve postoperatively regardless of the articulation, with no consistent or clinically meaningful differences between constructs [15,16,30,32,39]. Surgical technique and restoration of biomechanics remain key contributors to postoperative outcomes [35,37]. Systematic reviews highlight substantial variability in PROMs and follow-up intervals, limiting robust comparative interpretation [25,26,31].

Economic model-based analyses suggest potential cost implications of reduced instability with DM constructs. These findings are hypothesis-generating and sensitive to underlying assumptions, including local implant pricing and healthcare system context [9,10,22]. As such, this review should be interpreted as reflecting comparative clinical evidence and practice patterns rather than as definitive evidence of the superiority of one construct over the other.

Limitations and future directions

This study is limited by the observational, predominantly retrospective nature of the included evidence, including non-randomized implant allocation, heterogeneous follow-up durations, and variable outcome definitions. The inability to ascertain surgeon-level decision-making and to construct specific indications represents a major limitation. Implant-specific designs within the DM and CL categories were not analyzed separately, and inter-manufacturer variation may have influenced clinical outcomes. Key surgical variables, including surgical approach, component positioning, and soft-tissue repair techniques, were inconsistently reported, precluding assessment of their impact on instability and revision risk. Reporting of patient-reported outcomes, complication profiles, and economic endpoints was variable, limiting robust comparative and cost-effectiveness inferences.

Future research should prioritize prospective comparative studies with standardized reporting of time-to-event outcomes, complications, and contemporary patient-reported outcome measures. High-quality economic evaluations specific to revision THA are also needed to clarify cost-effectiveness and inform value-based implant selection. These measures will improve evidence quality, reduce residual confounding, and provide more definitive guidance on optimal articulation choice in complex revision settings.

## Conclusions

In revision THA, DM constructs are consistently associated with lower postoperative instability compared with CLs in comparative observational studies. However, these findings should be interpreted in the context of confounding by indication, as implant selection often reflects underlying case complexity rather than purely device performance. Evidence regarding reductions in aseptic re-revision remains heterogeneous, reflecting competing failure mechanisms and the limitations of non-randomized data. Economic implications remain uncertain due to the absence of primary cost-utility analyses. Implant selection should therefore be individualized, and high-quality prospective comparative studies with standardized outcome reporting are needed to define the optimal construct in revision THA.

## Appendices

Appendix A

Full Electronic Search Strategies

Databases searched: PubMed/MEDLINE (MEDical Literature Analysis and Retrieval System On-Line), Embase (via Elsevier), Scopus, Web of Science Core Collection, Cochrane Central Register of Controlled Trials (CENTRAL). Searches were conducted from January 2000 to 20 March 2025 and limited to human studies. No language restrictions were applied at the search stage. 

**Table 6:** Search Strategies Grey literature was searched to identify unpublished or ongoing studies using Google Scholar (first 1000 results screened using combinations of “dual mobility”, “constrained liner”, and “revision hip arthroplasty”)

Database	Search Strategy
PubMed (MEDLINE)	("Hip Prosthesis"[MeSH] OR "Hip Arthroplasty"[MeSH] OR "Total Hip Arthroplasty" OR "Revision Hip Arthroplasty" OR "Revision THA" OR "Revision Total Hip Arthroplasty") AND ("Dual Mobility" OR "Dual-Mobility" OR "Dual Mobility Cup") AND ("Constrained Liner" OR "Constrained Acetabular Liner" OR "Constrained Cup") AND ("Dislocation"[MeSH] OR Dislocation OR Instability OR "Revision Surgery" OR Re-revision)
Embase	('hip arthroplasty'/exp OR 'total hip arthroplasty' OR 'revision hip arthroplasty' OR 'revision total hip arthroplasty') AND ('dual mobility' OR 'dual mobility cup') AND ('constrained liner' OR 'constrained acetabular liner') AND ('hip dislocation'/exp OR dislocation OR instability OR 're-revision' OR 'revision surgery')
Scopus	TITLE-ABS-KEY (("revision total hip arthroplasty" OR "revision hip arthroplasty" OR "revision THA") AND ("dual mobility" OR "dual-mobility" OR "dual mobility cup") AND ("constrained liner" OR "constrained acetabular liner") AND (dislocation OR instability OR "re-revision"))
Web of Science Core Collection	TS=(("revision total hip arthroplasty" OR "revision hip arthroplasty" OR "revision THA") AND ("dual mobility" OR "dual-mobility" OR "dual mobility cup" OR "tripolar cup") AND ("constrained liner" OR "constrained acetabular liner") AND (dislocation OR instability OR "re-revision"))
Cochrane Library (CENTRAL)	("revision total hip arthroplasty" OR "revision hip arthroplasty") AND ("dual mobility" OR "dual-mobility") AND ("constrained liner" OR "constrained cup") AND (dislocation OR instability OR re-revision)

Appendix B

Model-Based Cost-Utility Scenario Analysis

A model-based cost-utility scenario analysis was conducted to explore the potential economic implications of dual-mobility (DM) constructs compared with constrained liners (CL) in revision total hip arthroplasty (rTHA). This analysis was informed by complication rates derived from the present systematic review and utilised cost and utility estimates from previously published economic evaluations [9,10,36].

This model is intended to provide contextual interpretation and should not be considered a definitive cost-effectiveness evaluation, as no included comparative study reported primary economic endpoints.

Model Structure

A decision-analytic framework was constructed comparing two strategies: (i) Revision THA using DM constructs and (ii) Revision THA using CLs. Three clinical outcome pathways were modelled: (i) Stable outcome, (ii) Postoperative instability (dislocation), and (iii) Aseptic re-revision. The time horizon was five years, aligned with mid-term survivorship reporting in the included studies. The analysis was conducted from a healthcare payer perspective.

Clinical Probabilities

Base-case probabilities were derived from aggregated event rates reported across the included comparative studies: (i) Dislocation: DM: 8%, CL: 16.1%; (ii) Aseptic re-revision: DM: 19.6%, CL: 22.7%. Where necessary, probabilities were applied assuming independence of events for modelling simplicity.

Cost Inputs

Cost parameters were extracted from published United States-based economic analyses: (i) Implant acquisition cost: DM: $4,300, CL: $2,800; (ii) Cost of managing dislocation: $8,000; (iii) Cost of re-revision surgery: $45,000. All cost estimates reflect reported values in the source publications [9,10,36].

Utility Inputs

Baseline health-related quality of life following stable revision THA was assumed equivalent between constructs. Utility decrements were applied for complication states based on published estimates: (i) Dislocation event: −0.1 QALY; (ii) Re-revision surgery: −0.2 QALY. These values were derived from previously published arthroplasty cost-utility literature [36].

Base-Case Analysis

Expected costs and quality-adjusted life-years (QALYs) were calculated for each strategy. Under base-case assumptions, DM constructs were associated with higher initial implant costs but lower expected complication-related costs, resulting in improved quality-adjusted survival.

The incremental cost-effectiveness ratio (ICER) for DM compared with CL was approximately $26,000 per QALY gained.

Sensitivity Analyses

Probabilistic sensitivity analysis was performed using 1,000 Monte Carlo simulations. Beta distributions were applied to clinical probabilities, and gamma distributions to cost parameters.

On the cost-effectiveness plane, most simulations fell in the northeast quadrant (higher cost, higher utility). At a willingness-to-pay (WTP) threshold of: $50,000 per QALY → DM had a 78% probability of being cost-effective; $100,000 per QALY → DM had a 92% probability of being cost-effective.

One-way sensitivity analysis demonstrated that results were most sensitive to: (i) the absolute difference in dislocation risk, and (ii) the implant acquisition cost differential. Under plausible parameter ranges derived from published literature, DM remained cost-effective across most scenarios.

Conclusion

Exploratory model-based economic analyses incorporating dislocation and aseptic re-revision probabilities derived from the included studies suggested potential downstream economic advantages for DM constructs at commonly accepted willingness-to-pay thresholds. These models estimated incremental cost-effectiveness ratios (ICER) of approximately $26,000 (£20,800) per QALY for DM constructs compared with $35,000-$48,000 (£28,000-£38,400) per QALY for CLs. DM constructs were associated with higher initial implant costs, but lower projected downstream costs driven by fewer instability-related complications and re-revisions. As no included study reported primary cost or cost-utility data, these findings should be interpreted as exploratory and hypothesis-generating.

Limitations

This analysis is model-based and not derived from primary economic data within the included comparative cohorts. Cost and utility inputs were obtained from external publications and may not be generalisable across healthcare systems. The model does not incorporate recurrent event cycles or long-term implant durability beyond the selected time horizon. Accordingly, findings should be interpreted as scenario-based estimates intended to contextualise complication-related economic impact rather than as definitive cost-effectiveness conclusions.
